# Breast Cancer Subtypes and Quantitative Magnetic Resonance Imaging: A Systemic Review

**DOI:** 10.3390/life12040490

**Published:** 2022-03-28

**Authors:** Toshiki Kazama, Taro Takahara, Jun Hashimoto

**Affiliations:** 1 Department of Diagnostic Radiology, Tokai University School of Medicine, Isehara 259-1193, Japan; junhashi@tokai-u.jp; 2 Department of Biomedical Engineering, Tokai University School of Engineering, Hiratsuka 259-1207, Japan; tt107100@tsc.u-tokai.ac.jp

**Keywords:** magnetic resonance imaging, dynamic contrast enhancement, diffusion weighted image, breast cancer, subtypes, quantitative values

## Abstract

Magnetic resonance imaging (MRI) is the most sensitive imaging modality for breast cancer detection. This systematic review investigated the role of quantitative MRI features in classifying molecular subtypes of breast cancer. We performed a literature search of articles published on the application of quantitative MRI features in invasive breast cancer molecular subtype classification in PubMed from 1 January 2002 to 30 September 2021. Of the 1275 studies identified, 106 studies with a total of 12,989 patients fulfilled the inclusion criteria. Bias was assessed based using the Quality Assessment of Diagnostic Studies. All studies were case-controlled and research-based. Most studies assessed quantitative MRI features using dynamic contrast-enhanced (DCE) kinetic features and apparent diffusion coefficient (ADC) values. We present a summary of the quantitative MRI features and their correlations with breast cancer subtypes. In DCE studies, conflicting results have been reported; therefore, we performed a meta-analysis. Significant differences in the time intensity curve patterns were observed between receptor statuses. In 10 studies, including a total of 1276 lesions, the pooled difference in proportions of type Ⅲ curves (wash-out) between oestrogen receptor-positive and -negative cancers was not significant (95% confidence interval (CI): [−0.10, 0.03]). In nine studies, including a total of 1070 lesions, the pooled difference in proportions of type 3 curves between human epidermal growth factor receptor 2-positive and -negative cancers was significant (95% CI: [0.01, 0.14]). In six studies including a total of 622 lesions, the pooled difference in proportions of type 3 curves between the high and low Ki-67 groups was significant (95% CI: [0.17, 0.44]). However, the type 3 curve itself is a nonspecific finding in breast cancer. Many studies have examined the relationship between mean ADC and breast cancer subtypes; however, the ADC values overlapped significantly between subtypes. The heterogeneity of ADC using kurtosis or difference, diffusion tensor imaging parameters, and relaxation time was reported recently with promising results; however, current evidence is limited, and further studies are required to explore these potential applications.

## 1. Introduction

Breast cancer is the most frequently diagnosed malignancy and the leading cause of cancer-related deaths among women [1]. Breast cancer is a heterogeneous disease with a high degree of diversity in the risks of therapeutic resistance and disease progression [2,3]. Therefore, individualized management is widely accepted [2,3]. However, previous classifications based on tumour size, grade, and histology cannot completely reflect tumour characteristics. Gene expression profiling has revealed four major breast cancer subtypes: luminal-A, luminal-B, human epidermal growth factor receptor 2 (HER2)-enriched, and basal-like [3]. Each subtype has varied prognoses, progression risks, responses to treatment, and survival outcomes. Commercial multigene assays are expensive and time-consuming; thus, the St. Gallen International Expert Consensus panel has suggested surrogate subtypes based on semiquantitative immunohistochemical scoring of oestrogen receptor (ER) and progesterone receptor (PR) status, in situ hybridisation tests for HER2 overexpression, and proliferation according to the Ki-67 labelling index (Ki-67) [2,3,4].

Luminal-type (hormone receptor [HR]-, ER- or PR-positive) breast cancer is the most frequent type and is divided into luminal-A and luminal-B subtypes, which are defined by low- (Ki-67 < 14) and high- (HER2-positive or Ki-67 ≥14) proliferation subtypes [2,3,5]. Endocrine therapy is the mainstay of systemic therapy. Luminal-A is not responsive to chemotherapy, while luminal-B may be amenable to chemotherapy as well as endocrine therapy [2,3].

HR-negative breast cancers include HER2-positive and basal-like subtypes. They tend to be of higher grades with higher Ki-67 indexes [6,7,8,9,10,11]. HER2, a transmembrane receptor tyrosine kinase in the epidermal growth factor receptor family, is amplified or overexpressed in approximately 20% of breast cancers and is associated with poor prognosis, although good response to HER2-targeted therapies [4,6].

Basal-like breast cancers on multigene assays are usually triple-negative (TN) breast cancers on semiquantitative scoring [11]. TN breast cancer is more widely used than basal-like cancer. It accounts for approximately 15% of all breast tumours. TN breast cancer is characterised by a lack of ER, PR, or HER2 expression. TN tumours are usually high-grade invasive ductal carcinomas with a high risk of distant relapse in the first 3–5 years following diagnosis [11]. Chemotherapy is the standard systemic therapy for TN breast cancers [4,11].

Local therapy for all patients with non-metastatic breast cancer consists of surgical resection, with consideration of postoperative radiation. The choice of systemic therapy is determined by the cancer subtype [4]. While the breast cancer subtype is diagnosed by immunochemical staining of biopsied or resected specimens, receptor expression can change during treatment [12,13]. In approximately 25% of cases, the HER2 status may be discordant between the primary tumour and metastases [12,13]. Loss of HER2 status after neoadjuvant chemotherapy has also been reported [13]. In patients with recurrent or metastatic lesions, multiple biopsies may be desirable; however, biopsy is an invasive and costly procedure. Non-invasive assessment of receptor status, especially in recurrent lesions, may improve personalised treatment.

Breast magnetic resonance imaging (MRI) offers information on not only the cross-sectional morphology of the lesion but also functional lesion features [14,15]. Researchers have studied the relationship between quantitative MRI findings and breast cancer subtypes. This systematic review aimed to evaluate the relationship between quantitative MRI findings and breast cancer subtypes to determine whether MRI findings can predict breast cancer subtypes.

## 2. Materials and Methods

### 2.1. Protocol Registration

This systematic review was conducted according to the pertinent sections presented in the Preferred Reporting Items for Systematic Reviews and Meta-Analyses (PRISMA) statement, which was used for the analysis [16]. The protocol was registered in the International Prospective Register of Systematic Reviews (PROSPERO) database with a registration number of 308,403 (https://www.crd.york.ac.uk/PROSPERO, accessed on 16 March 2022).

### 2.2. Search Strategy

The PubMed database was screened for studies on the associations between quantitative MRI values and breast cancer. We limited the literature search to articles published from 1 January 2002 to 30 September 2021. One reviewer performed the data acquisition using the following search terms: “magnetic resonance imaging”, “breast neoplasms” and “subtype or phenotype or Ki-67 or receptors, oestrogen or receptors, progesterone or receptor, and ErbB-2”. The secondary references were manually checked and included in the study.

### 2.3. Inclusion and Exclusion Criteria

**Figure 1:**
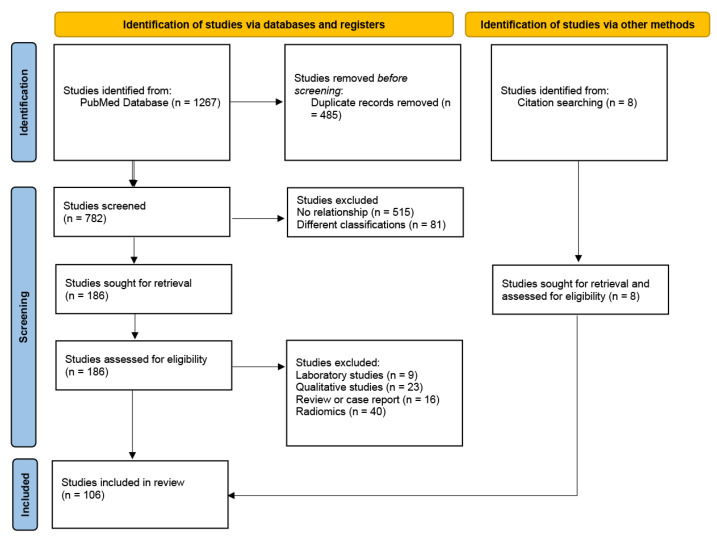
Flow diagram for literature search.

The primary endpoint of the systematic review was the association between molecular subtypes of breast cancer and quantitative MRI values. Studies (or subsets of studies) were included if they satisfied all the following criteria: (1) inclusion of patients with invasive breast cancer confirmed by histopathology, (2) pre-treatment MRI, (3) quantitative analysis of MRI, (4) MRI correlation with breast cancer subtypes or factors that determine subtype, (5) human women, and (6) English language. We considered studies reporting visual evaluations, such as high signal intensity and heterogeneity without quantification, to be qualitative studies and did not include them. The exclusion criteria were: (1) reviews without meta-analyses, case reports, or editorials, and (2) radiomics, machine learning, or artificial intelligence studies. We regarded simple methods, such as histogram analysis, diffusion tensor imaging (DTI), and pharmacokinetic analysis, as non-radiomics. The results of this study are summarised in Figure 1. Ethical approval was not required for this study.

### 2.4. Article Selection and Data Extraction

One radiologist screened the selected titles and abstracts to ensure conformity with the inclusion criteria and documented the rationale for exclusion. Supplementation for article selection was performed by screening the reference lists. After screening, the full texts were reviewed. The following data were extracted from the literature: authors, year of publication, number of patients, number of cases in each subtype, sequences and analytic methods included in image analysis, and results.

### 2.5. Quality Assessment

The methodological quality of the acquired studies was checked based on the Quality Assessment of Diagnostic Studies (QUADAS 2) [17].

### 2.6. Data Synthesis

When there were more than five studies with similar methodologies, conflicting results, and no prior meta-analysis, a meta-analysis was performed using RevMan v5.4 (Cochrane Collaboration, London, UK). The mean difference in the prevalence of imaging findings was analysed using a random effects model. In the analysis of the apparent diffusion coefficient (ADC), we suspected that region-of-interest (ROI) placement might be a cause of heterogeneous results; therefore, we identified the ROI placement methods and classified them.

## 3. Results

### 3.1. Literature Search

A total of 1267 articles were identified in the electronic databases. Following the removal of 485 duplicates, the titles and abstracts of 782 articles were screened. Of the 782 articles, 596 did not fulfil the inclusion criteria. Nine laboratory studies, 23 qualitative studies, 16 reviews or case reports, and 40 studies with radiomics were excluded. Eight studies were identified from the citation search and were also included. Figure 1 shows an overview of the literature search and study selection process.

### 3.2. Study Characteristics

The included studies encompassed 104 original studies and two meta-analyses, with publication years ranging from 2003 to 2021. A total of 12,989 patients, excluding double-counted patients in prior meta-analyses (n = 3466), met the selection criteria. Quantitative features were mostly derived from kinetic parameters measured using DCE (n = 45) or diffusion-weighted imaging (DWI) (n = 68). Five studies assessed relaxation time and four assessed magnetic resonance spectroscopy (MRS). Of these, 10 studies analysed both DWI and DCE, one DCE and relaxation time, one DCE and MRS, one DCE, DWI, and relaxation time, and one DCE, DWI, and MRS.

### 3.3. Methodological Quality of the Included Studies

Patient selection was generally well-defined within the respective methodology. However, in 18 studies, more than 10% of cases were excluded for ambiguous reasons such as poor image quality. In one study, the sum of cases did not match the total number. This may have contributed to potential bias. All studies reported the methodology of the index test and were, thus, not considered a significant source of potential bias. Although immunohistochemical staining criteria differed among studies, the reference standards in all studies were histopathology with immunohistochemical staining and were not considered a significant source of potential bias. The subtype classification method was adapted from the 2011 St. Gallen Consensus meeting [18]. All patients underwent the reference test with the appropriate timing when they were included in the analysis.

### 3.4. Dynamic Contrast-Enhanced-Magnetic Resonance Imaging

Investigations regarding DCE-MRI are summarised in Table 1. DCE-MRI offers information not only on lesion cross-sectional morphology, but also on functional lesion features, such as tissue perfusion and enhancement kinetics [14]. In DCE-MRI, highly vascularised tumours tend to show early and strong contrast enhancement and wash-out of contrast in the delayed phase. Many methods have been proposed, with the proportion of time-intensity curve patterns (n = 14) being the most widely used.

**Figure 2:**
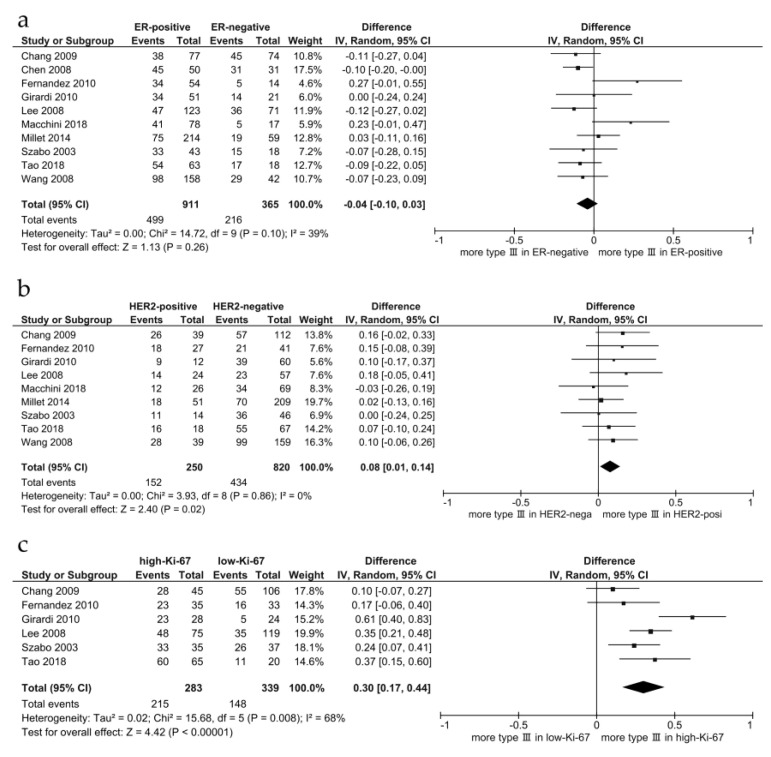
Forest plots of the pooled difference in proportions of type Ⅲ (wash-out) curves between ER-positive and ER-negative cancers (**a**), between HER2-positive and -negative cancers (**b**), and between the high- and the low-Ki-67 groups (**c**).

Twelve studies evaluated time-intensity curve patterns and ER status; however, two studies did not show the exact values and were excluded from this meta-analysis [50,53]. In the meta-analysis of these 10 studies, including a total of 1276 lesions [19,20,21,23,24,25,30,43,46,47], the pooled difference in proportions of type Ⅲ curves (wash-out) between ER-positive cancer and ER-negative cancers for all included tumours was −0.04, (95% confidence interval [CI] = [−0.10, 0.03]), heterogeneity τ^2^ = 0.00, I^2^ = 39%, test for overall effect Z = 1.13 (*p* = 0.26) (Figure 2a).

Ten studies evaluated time-intensity curve patterns and HER2 status; however, one study did not mention the exact values and was excluded from this meta-analysis [53]. In the meta-analysis of these nine studies, including a total of 1070 lesions [19,20,23,24,25,30,43,46,47], the pooled difference in proportions of type Ⅲ curves (wash-out) between and HER2-positive and HER2-negative cancers for all included tumours was 0.08, (95% CI = [0.01, 0.14]), heterogeneity τ^2^ = 0.00, I^2^ = 0%, test for overall effect Z = 2.40 (*p* = 0.02) (Figure 2b).

Seven studies evaluated time-intensity curve patterns and Ki-67 status; however, one study did not mention the exact values and was excluded from this meta-analysis [20]. In the meta-analysis of these six studies including a total of 622 lesions [21,22,23,24,25,26], the pooled difference in proportions of type Ⅲ curves (wash-out) between the high- and low-Ki-67 groups for all included tumours was 0.30, (95% CI = [0.17, 0.44]), heterogeneity τ^2^ = 0.02, I^2^ = 68%, test for overall effect Z = 4.42 (*p* < 0.01) (Figure 2c).

Seventeen studies evaluated the enhancement ratio, and conflicting results were reported. However, highly variable analyses among the studies prevented us from conducting a meta-analysis [19,20,23,25,29,30,31,32,35,40,41,42,43,44,46,49,64].

A pharmacokinetic analysis was performed in 11 studies. Ktrans is a transfer constant that measures the rate of transport of contrast medium from the plasma to the extravascular extracellular space (EES), and provides a measure of vascular permeability and blood flow. Ve is the tumour volume occupied by the EES and Kep describes the outflow rate of the contrast medium from the EES back to the plasma. Higher Kep and lower Ve values in DCE-MRI were observed in the TN subtype [27,28]. Two studies analysed the relationship between TN cancers and pharmacokinetic parameters. Both reported significantly higher Kep and lower Ve values in TN cancers than in other subtypes [27,28]. However, the relationships between other pharmacokinetic parameters and prognostic factors were conflicting. Six studies evaluated whether HER2-positive cancers had a higher Ktrans than that in HER2-negative cancers. Two of them demonstrated significant differences [38,61], and the other four demonstrated no significant differences [28,37,39,48]. Similarly, eight studies evaluated the relationship between Ki-67 status and Ktrans. Three studies demonstrated significant differences, and five studies demonstrated no significant differences [28,34,37,38,39,48,56,61]. These studies used the same model proposed by Tofts [65]; however, there were highly variable values between the studies, which hindered the meta-analysis. For example, the mean Ktrans of invasive breast cancers with a low Ki-67 (Ki-67 < 14 %) in one study was 2.56/min [61], whereas that in another study was 0.18/min [38].

Two studies reported that the HER2 subtype exhibited higher rapid early contrast uptake [36,42]. Many other indices have been proposed; however, these have been evaluated in only a few studies or conflicting results were reported. For example, two studies reported that a short peak time was associated with positive HER2 status [19,24]; however, three did not find any significant differences [20,30,32].

Three studies evaluated background parenchymal enhancement (BPE) and breast cancer subtypes [54,55,63]. One study reported that moderate and marked BPE prevailed over minimal and mild BPE in patients with TN cancers [54], whereas another reported that BPE was significantly lower in patients with TN cancer compared with patients with non-TN cancers [63].

### 3.5. Diffusion-Weighted MR Images

#### 3.5.1. Apparent Diffusion Coefficient

The DWI results are summarised in Table 2. Sixty-three of the 68 DWI studies analysed the ADC. There have been two meta-analyses regarding the subtypes and Ki-67 [66,67]. Meyers et al. reported that the ADC values of breast cancer subtypes overlapped significantly, with no clear proposed threshold to distinguish between them [66]. In this meta-analysis, the I^2^ ranged from 95% to 98%, suggesting considerable heterogeneity. Surov et al. reported that correlation coefficient of ADC and Ki-67 was −0.22 (95% CI = [−0.50; 0.06]) with an I^2^ of 91%, suggesting considerable heterogeneity [67].

These meta-analyses may be affected by heterogeneous methodologies, one of which might be the ROI placement. We classified ROI placement as follows: evaluation of the whole lesion (whole), solid portion of the lesion excluding cystic/necrotic/haemorrhagic portion (solid), and ROI placement methodology not found (unknown). One study evaluated the effect of ROI placement using both solid and whole methods [108]. Seventeen studies with a known ROI placement methodology evaluated whether TN breast cancers had higher ADC values than other cancer subtypes. In the solid portion measurement group, one of the 12 studies demonstrated significant differences [50,52,53,71,72,74,79,82,86,91,105,110], while four of the five studies reported significant differences in the whole lesion measurement group [29,51,78,80,96]. Twelve studies with known ROI placement methodology evaluated whether luminal-B-type breast cancers had lower ADC values than luminal-A-type cancers. In the solid portion measurement group, six out of nine studies demonstrated significant differences [41,50,77,79,85,97,105,110,117], while one out of three studies demonstrated significant differences in the whole lesion measurement group [78,80,98].

Although ADC values differed among breast cancer subtypes, the ADC values of different tumour subtypes overlapped significantly [66,110]. Instead of using the mean ADC, more sophisticated methods, such as differences in ADC and diffusion kurtosis, have been evaluated with promising results. Two studies evaluated the relationship between ADC differences (maximum ADC to minimum ADC) and subtypes. They reported that the ADC difference was significantly associated with Ki-67 expression [98,111].

In probability theory and statistics, the alteration of a normative distribution pattern is known as kurtosis. Diffusion kurtosis imaging attempts to account for this variation to provide a more accurate model of diffusion as a reflective marker for tissue heterogeneity [119]. Similarly, skewness, which reflects the asymmetry of ADC value distribution, has been introduced in cancer imaging [105,118]. Three studies reported a positive association between diffusion kurtosis and the Ki-67 index [81,107,109]. Similarly, one study reported significantly higher ADC kurtosis in the TN group than in the ER-positive group [96].

#### 3.5.2. Intravoxel Incoherent Motion

Intravoxel incoherent motion (IVIM) MRI is a non-invasive imaging method that allows the evaluation of both tissue diffusivity and tissue microcapillary perfusion. When DWI is performed with multiple b-values (usually 0–1000 smm^−2^), the signal intensity at low b-values (e.g., 0–100 smm^−2^) reflects both water diffusion in tissues and microcirculation within the capillaries. In contrast, at higher b-values, the signal intensity is more reflective of tissue diffusivity. Thus, the classical IVIM model uses a biexponential analysis that provides the tissue diffusion coefficient (Dt), perfusion-related diffusion (Df), and perfusion fraction (f).

Ten studies evaluated the association between IVIM and breast cancer subtypes. Seven studies evaluated whether high-Ki-67 tumours had lower Dt than low-Ki-67 tumours. Three of them demonstrated significant differences [41,89,104], whereas the other four demonstrated no significant differences [8,64,93,115]. Five studies evaluated whether HER2-positive cancers had higher Df than HER2-negative cancers. Two demonstrated significant differences [89,115], and the other three demonstrated no significant differences [8,52,93]. Owing to the large heterogeneity of the results, we did not perform a meta-analysis.

#### 3.5.3. Diffusion Tensor Imaging

DTI is a conceptual framework that provides quantitative information on the directional diffusivity of water molecules [120]. The measurement of DTI indices, such as ADC, fractional anisotropy (FA), mean diffusivity (MD), radial diffusivity (RD), geodesic anisotropy (GA), relative anisotropy (RA), and volume ratio (VR), provides quantification. The mammary ductal network may result in diffusion anisotropy in healthy fibroglandular tissue [121]; however, cancer cells may destroy these structures, leading to reduced anisotropy. Two studies reported that FA was significantly higher in the low-Ki-67 group and ER-positive cancers [92,94].

### 3.6. Relaxation Time

The relaxation time findings are summarised in Table 3. One study reported significantly longer T2* relaxation times in higher histologic grades, which correlated with high signal intensity on T2-weighted imaging [122]. Using synthetic MRI, three studies assessed T1 and T2 relaxation times [57,61,123]. Two reported significantly higher T2 in the HR-negative group compared to the HR-positive group [61,123].

### 3.7. Magnetic Resonance Spectroscopy

The MRS findings are summarised in Table 4. MRS provides biochemical information regarding the investigated tissues. Increased choline (Cho) is a marker of elevated cellular proliferation rates in breast cancer [125]. Four studies evaluated the relationship between MRS and subtypes [21,50,126,127]. Conflicting results were reported with TN breast cancers and MRS [50,126].

## 4. Discussion

### 4.1. DCE-MRI

DCE-MRI is a standard diagnostic technique with high sensitivity and variable specificity for characterising breast lesions. Angiogenesis is one of the main factors affecting gadolinium uptake and contributes to internal enhancement patterns and kinetic curves. In DCE-MRI, highly vascularised tumours tend to show early and strong contrast enhancement and wash-out of contrast in the delayed phase. This study demonstrated that a significantly higher proportion of type 3 curves was observed in the high-Ki-67 group compared with the low-Ki-67 group. This finding was consistent with the correlation between vascular endothelial growth factor (VEGF) and histological grade reported in a pathological study [128].

In the meta-analysis, a significantly higher proportion of type 3 curves was observed in HER2-positive cancers than in HER2-negative cancers. This finding was consistent with the correlation between the overexpression of VEGF and HER2-positive tumours in pathological studies [6,7,8,22,36].

Although a negative correlation between ER status and cytosolic levels of VEGF has been reported in pathological studies [128] and the proportion of type 3 curves tended to be lower in ER-positive cancers, no significant difference was observed in this meta-analysis.

However, the wash-out curve itself is a common finding in breast cancer, and the prediction of subtypes based on this finding is difficult. Many indices have been proposed; however, other indices are immature and conflicting results have been reported.

### 4.2. DWI

DWI detects the Brownian motion of water protons, thereby reflecting the biological characteristics of the tissue. ADC is used to quantify Brownian motion. By imaging alterations in the microscopic motion of water molecules, DWI can yield novel quantitative and qualitative information reflecting cellular changes that can provide unique insights into tumour cellularity, with a potential role in the characterisation of breast masses [129]. The decreased ADC values in malignant tumours may be due to their increased cellularity, larger nuclei with more abundant macromolecular proteins, and reduced extracellular space. These tissue factors hinder proton diffusion and, consequently, lower ADC values [66,130].

Higher Ki-67 expression usually implies rapid proliferation, and consequently, increased cellularity, which restricts the diffusion of water molecules in the extracellular and extravascular spaces and is presumed to cause reduced ADC values [131]. A weak inverse correlation between tumour cellularity and ADC values has been described, and further associations between proliferation rate and tumour aggressiveness have been proposed [67,77,129]. 

However, several studies have suggested that highly aggressive invasive breast cancers rapidly outgrow their vascular supply in certain areas, leading to prolonged hypoxia within the tumour and subsequent necrosis [106,132,133,134]. Areas of intratumoral necrotic tissue and loss of cell membrane integrity are associated with increased intratumoral water diffusion. This may explain the higher ADC value in the TN subtype when the entire lesion ADC is measured [78,135,136].

Neoangiogenesis is the basis of cancer cell proliferation. Pathological studies have demonstrated an association between cytosolic levels of VEGF, an angiogenesis stimulator, and histologic grade, as well as a negative correlation with ER status [6,7,8,22,36,128]. Owing to the perfusion effect, high vascularity can result in increased ADC values. Furthermore, tumour vessels tend to have larger diameters than normal microvessels as well as discontinuities in the vascular walls, leading to increased total extracellular fluid volumes. The higher tumour blood flow and increased extracellular fluid appear to compensate for the low ADC of high cellularity [37,73,78,80,101,112,137,138].

These paradoxical phenomena may cause confusion in subtype predictions based on the mean ADC. Meyer et al. reported in a meta-analysis that ADC values cannot discriminate immunohistochemical molecular subtypes [66]. To overcome the increased ADC by necrosis, methods of assessing heterogeneity using ADC kurtosis and ADC differences, which may reflect high cellular areas and necrosis, have been proposed, with promising results [63,96,98,107,111,118].

### 4.3. Relaxation Time and T2-Weighted Images

**Figure 3:**
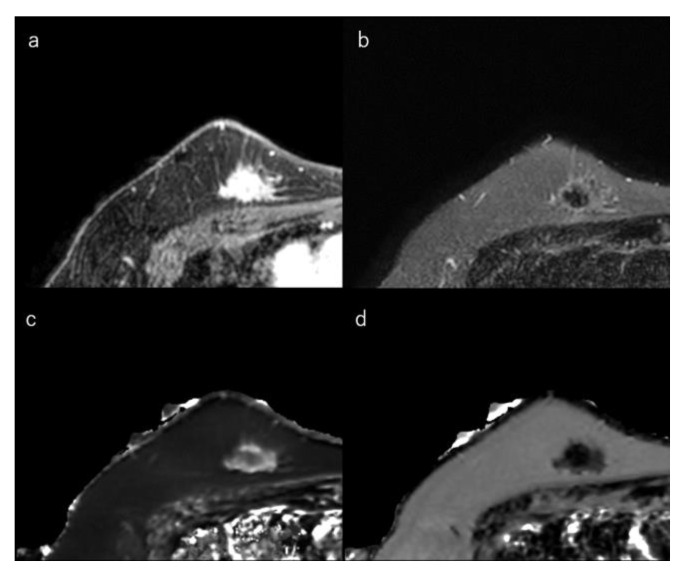
(**a**) Dynamic contrast-enhanced MR image in a 50-year-old woman with luminal-A type breast cancer shows a spiculated mass. (**b**) Short-tau inversion recovery image shows a low signal mass. (**c**) T1 map (window width/centre = 1400/2400 ms) shows an intermediate signal mass; Mean T1 of the mass is 986 ms. (**d**) T2 map (window width/centre = 160/240 ms) shows a low signal mass; mean T2 of the mass is 62 ms.

In general, HR-positive tumours, which often have low proliferation, may demonstrate stromal reactions and fibrosis [139]. Intratumoral iso/low T2-signal intensity is a feature of breast cancer, which may reflect this fibrosis (Figure 3) [14,140,141,142,143]. In contrast, TN breast cancers have high signal intensity on T2-weighted images owing to necrosis [29,144,145,146]. In addition, a higher tumour grade often correlates with higher neoangiogenesis [128]. Angiogenesis increases total extracellular fluid volume and oedema. Thus, high-grade tumours may demonstrate high signal intensity on T2-weighted images. High signal intensity on T2-weighted images is also correlated with tumour grade [106,147,148,149].

These studies involved subjective qualitative analyses, which makes it difficult to apply their results in clinical practice to assess the HR status or subgroup categorisation of ER-positive breast cancers. Seo et al. reported significantly longer T2 * relaxation times in higher histological grades [122]. Recent advances in quantitative MRI have enabled the acquisition of both MR images and quantitative MR data in a single scan [57,150]. Synthetic MRI enables us to appreciate subtle quantitative MRI value differences that are invisible to radiologists’ eyes alone. Synthetic MRI can also measure T1 values and proton density, which cannot be assessed using T2-weighted images [150,151,152]. Contrary to the ADC, T2 values of highly proliferative tumours were higher than those of low proliferative tumours [61,123]. Because these value assessments do not experience a paradoxical phenomenon, they may be more useful than ADC. In a radiomics study, Liang et al. reported that a T2-weighted image-based radiomics classifier was a significant predictor of Ki-67 status in patients with breast cancer, whereas contrast-enhanced image-based classifiers failed to discriminate in the validation dataset [153].

### 4.4. Luminal-Type Breast Cancer

This section explains the MRI characteristics of the breast cancer subtypes. In general, HR-positive tumours demonstrated stromal reaction, fibrosis, and perilesional spiculations [139]. An irregular mass margin and a non-round shape were significantly associated with luminal-A-type cancers [32,140,154]. Intratumoral iso/low T2-signal intensity may reflect fibrosis and is also associated with the luminal subtypes [14,140,141,142]. Multifocal or multicentric carcinoma is less common in the luminal-A type than in the luminal-B or HER2 types [140,155]. Compared to the other subtypes, luminal-A-type breast cancers tended to show less strong enhancement [35]. Kato et al. reported that rim enhancement occurred significantly less frequently in luminal-A-type breast cancers [85] (Figure 3).

**Figure 4:**
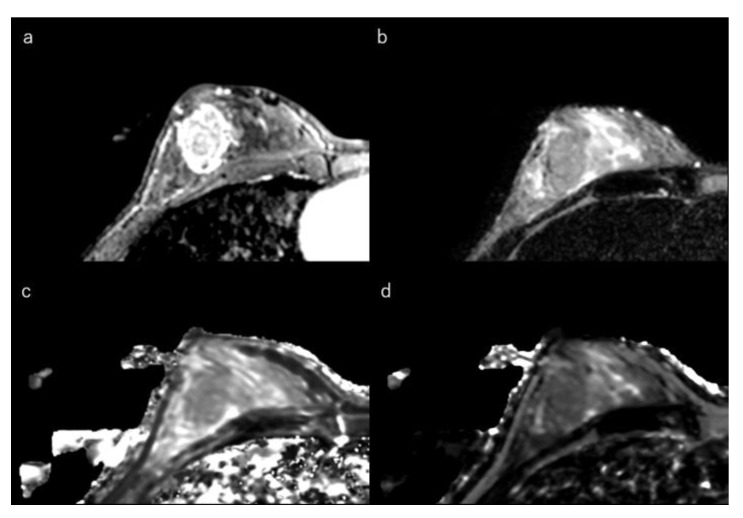
(**a**) Dynamic contrast-enhanced MR image in a 32-year-old woman with luminal-B type breast cancer shows a heterogeneously enhanced oval mass with rim enhancement. (**b**) Short-tau inversion recovery image shows an intermediate signal mass. (**c**) T1 map (window width/centre = 1400/2400 ms) shows an intermediate signal mass; Mean T1 of the mass is 1174 ms on T1 map. (**d**) T2 map (window width/centre = 160/240 ms) shows an intermediate signal mass; mean T2 of the mass is 97 ms on T2 map.

Tumour roundness is positively correlated with Ki-67 index [154]. Luminal-B subtypes are more often associated with multicentric/multifocal disease than are luminal-A cancers [32,156,157] and are also enriched for fibroblast growth factor receptor gene amplification, which has been implicated in angiogenesis [33,158]. This may lead to a higher ratio of lesion enhancement on DCE-MRI and heterogeneous internal enhancement [32,33,41,159] (Figure 4).

### 4.5. HER2-Enriched Subtype

**Figure 5:**
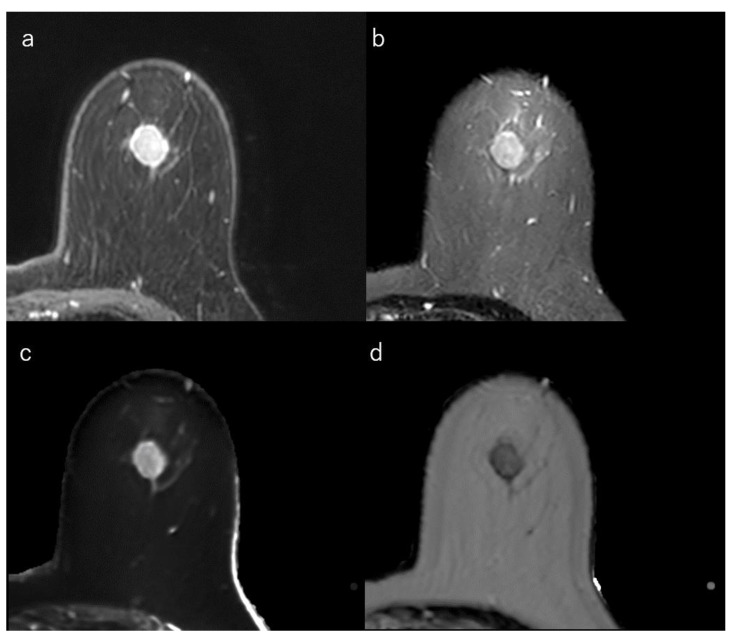
(**a**) Dynamic contrast-enhanced MR image in a 56-year-old woman with human epidermal growth factor receptor 2-enriched breast cancer shows a round mass. (**b**) Short-tau inversion recovery image shows a high signal mass. (**c**) T1 map (window width/centre = 1400/2400 ms) shows a high signal mass; mean T1 of the mass is 1256 ms, (**d**) T2 map (window width/centre = 160/240 ms) shows an intermediate signal mass; and mean T2 of the mass is 88 ms.

HER2, a transmembrane receptor tyrosine kinase in the epidermal growth factor receptor family, is amplified or overexpressed in approximately 20% of breast cancers and is associated with poor prognosis, although it responds well to HER2-targeted therapies [4,6]. The cellular-level effects of HER2 overexpression include increased cell proliferation, cell survival, mobility, and invasiveness, as well as neo-angiogenesis by increasing VEGF production [6,7,8]. On gross pathology, a smooth mass margin was associated with the HER2-enriched subtype [32] (Figure 5). The presence of microcalcifications, especially branching or fine linear morphology, was associated with mammography [6]. HER2-enriched subtypes are more often associated with multicentric/multifocal disease than luminal-A cancers [32,156,157,160]. Increased angiogenesis in the HER2-enriched subtype leads to rapid early contrast uptake and a higher proportion of wash-out curves on DCE-MRI (Figure 2) [36].

### 4.6. TN Breast Cancer

**Figure 6:**
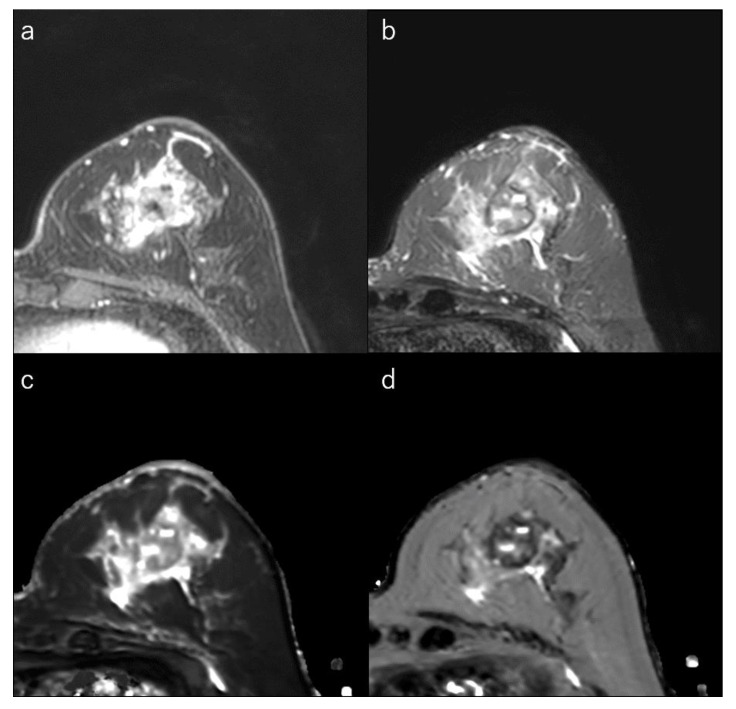
(**a**) Dynamic contrast-enhanced MR image in a 73-year-old woman with triple-negative breast cancer shows an irregular mass with rim enhancement. (**b**) Short-tau inversion recovery image shows focal areas of very high signal within the mass. (**c**) T1 map (window width/centre = 1400/2400 ms) shows focal very high signals; Mean T1 of the mass is 1533 ms. (**d**) T2 map (window width/centre = 160/240 ms) shows focal very high signals within the mass; and mean T2 of the mass is 113 ms.

TN breast cancer is highly associated with the presence of a central scar, tumour necrosis, the presence of spindle cells or squamous metaplasia, high total mitotic count, and high nuclear-cytoplasmic ratio [9,10,145]. These cancers are also more likely to show round, oval, or lobulated masses and are more likely to be unifocal compared to ER+/PR+/HER2 tumours [29,145,146,161,162]. MRI often shows areas of intratumoral high T2 signal intensity, lobulated shape, rim enhancement, and smooth margins (Figure 6) [29,144,145,146,162]. The rim enhancement can be explained by high angiogenesis in the periphery of the tumour. Very high intratumoral signal intensity on T2-weighted MR images and an elongated T2 relaxation time may be associated with intratumoral necrosis [29,123,145]. When the necrotic areas are included, the ADCs of TN cancers are higher than of luminal-type breast cancers [29,51,78,96].

### 4.7. Limitations

A major limitation of this review was the exclusion of complex radiomics studies. Forty studies reporting radiomics in breast MRI and breast cancer subtypes were excluded from this analysis (Figure 1). Several radiomics methods have been proposed, with promising results. Further studies, including systemic reviews in this field, are warranted. This meta-analysis only included studies published in English, resulting in selection bias, as the data might not be representative of the non-native English-speaking regions of the world.

## 5. Conclusions

Conventional quantitative MRI features, such as the time-intensity curve and mean ADC, might play a limited role in the prediction of breast cancer subtypes. While ROI placement is essential for quantitative analysis, it currently depends on the radiologists. Aggressive breast cancers, especially the TN subtype, contain necrosis, which causes heterogeneity within the tumour. Sophisticated evaluation of tumour heterogeneity, further research of recently introduced techniques, and standardised interpretation of MR images may improve non-invasive breast cancer subtype classification and personalised treatment for patients with breast cancer.

## Figures and Tables

**Table 1:** Summary of dynamic contrast-enhanced MRI findings according to molecular prognostic factors and subtypes, arranged in chronological order based on publication data in PubMed.

Author, Year	Number of Breast Ca (Subtypes)	Assessment	Findings
Szaboet al., 2003 [19]	61	Time to peak, TIC, enhancement ratio	Short time to peak associated with HER2-positive status and ER-negative status. Type III curves (wash-out) associated with Ki-67 positivity.
Lee et al., 2008 [20]	194	TIC, enhancement ratio	Washout curve may predict a higher level of Ki-67.
Chen et al., 2008 [21]	90	TIC	No significant association between kinetic parameters and ER status.
Makkat et al., 2008 [22]	55	Deconvolution	Higher tumour blood flow in PR negative group than in PR-positive group.
Girardi et al., 2010 [23]	72	TIC, enhancement ratio	Significant correlation between Ki-67 and type III curves (wash-out).
Chang et al., 2009 [24]	139	Time to peak, TIC	Short time to peak correlated with ER-negative status.
Fernández-Guinea et al., 2010 [25]	68	Time to peak, TIC, enhancement ratio	Short time to peak associated with Ki-67.
Li et al., 2010 [26]	31	T2 * dynamic, Ktrans, Kep, Ve, MTT	R2* influenced by blood volume in breast carcinomas.
Li et al., 2010 [27]	37 (16T N, 21 L)	Ktrans, Kep, Ve, MTT	Lower Ve, shorter MTT and higher Kep in TN than those in non-TN.
Koo et al., 2012 [28]	70	Ktrans, Kep, Ve	Higher Ktrans and Kep in ER-negative group than those in ER-positive group. Lower Ve in ER-negative group than in ER-positive group.
Youk et al., 2012 [29]	271	TIC, enhancement ratio	No specific kinetic feature for TN.
Millet et al., 2014 [30]	273	Initial enhancement, time to peak, enhancement ratio	No significant association between kinetic parameters and either HR or HER2.
Yamaguchi et al., 2015 [31]	192	Percent volume of TIC, enhancement ratio	No significant association between kinetic parameters and either HR or HER2.
Kawashima et al., 2014 [32]	116 (24 LA, 29 LB, 23 HER2, 40 TN)	Enhancement ratio, TIC	Higher enhancement ratio at 2 min in LB and HER2 than in LA.
Mazurowski et al., 2014 [33]	48 (28 LA, 8 LB, 4 HER2, 8 TN)	Lesion enhancement rate to background parenchymal enhancement rate	Cancers with higher ratios of lesion enhancement rate to background parenchymal enhancement rate were more likely to be LB.
Li et al., 2015 [34]	52	Ktrans, Kep, Ve	No significant association between kinetic parameters and Ki-67.
Leong et al., 2015 [35]	194 (140 L, 18 HER2, 36 TN)	Enhancement ratio, volumetric analysis of the kinetic patterns, TIC	Higher enhancement ratio and rapid initial enhancement in ER-negative, PR-negative, and TN. Higher rapid washout component in HER2-positive group than in HER2-negative group.
Blaschke et al., 2015 [36]	112 (73 L, 11 HER2, 28 TN)	percent volume of enhancement	Greater percent volume for HER2 subtype in the early phase compared to L and TN.
Lee et al., 2016 [37]	52 (39 L, 4 HER2, 9 TN)	Ktrans, Kep, Ve, initial AUC	Median Ve higher in PR-positive group than in the PR-negative group.
Shin et al., 2017 [38]	88 (39 LA, 49 LB)	Ktrans, Kep, Ve	Higher Ktrans in LB than in LA.
Catalano et al., 2017 [39]	21 (6 LA, 8 LB, 7 HER2)	Ktrans, Kep, Ve	Higher Kep in non-L than in L. Higher Kep in HER2-positive group than in HER2-negative group.
Caiazzo et al., 2018 [40]	27	Enhancement ratio, maximum enhancement, slope of the enhancement	Positive correlation between Ki-67 and both maximum enhancement and maximum slope of the enhancement.
Kawashima et al., 2017 [41]	137 (82 LA, 55 LB)	Enhancement ratio, SER	Higher SER in LB than in LA.
Trimboli et al., 2018 [42]	25	Time to peak, maximum enhancement, enhancement ratio, TIC	Enhancement ratio correlated with HER2 overexpression.
Wang et al., 2018 [43]	116 (43 LA, 55 LB, 7 HER2, 11 TN)	TIC, time to peak, enhancement ratio	HER2 status associated with type III curves (wash-out). LA less likely to have type III curves (wash-out).
Heacock et al., 2018 [44]	142 (83 L, 31 HER2, 28 TN)	initial enhancement ratio	Higher initial enhancement ratio in HER2 subtype and TN compared to L.
Incoronato et al., 2018 [45]	49 (13 LA, 29 LB, 4 HER2, 3 TN)	Ktrans, Kep	Kep_max_ could discriminate between LA and LB subtypes. Ktrans_max_ could discriminate between LA and non-L subtypes.
Macchini et al., 2018 [46]	95 (24 LA, 54 LB, 5 HER2, 12 TN)	TIC, enhancement ratio, maximum enhancement, AUC, maximum slope, wash-out rate, time to peak	Subtypes related to maximum enhancement, peak time, and maximum slope. ER correlated with maximum and relative enhancement, wash-in rate, and AUC.
Tao et al., 2018 [47]	85 (67 L, 18 others)	TIC	Distribution of curve types differed significantly for ER and Ki-67 but not PR or HER2 expression.
Nagasaka et al., 2019 [48]	101 (82 L, 19 others)	Ktrans, Kep, Ve	Lower Ve in the high Ki-67 group.
Gigli et al., 2019 [49]	75 (30 TN, 45 others)	Peak enhancement, time to peak, SER, enhancement ratio	Lower enhancement ratio and higher SER in TN.
Montemezzi et al., 2018 [50]	453 (66 LA, 292 LB, 39 HER2, 56 TN)	TIC	Smaller proportion of type III curves (wash-out) in LA.
Xie et al., 2019 [51]	134 (26 LA, 68 LB, 18 HER2, 22 TN)	Maximum slope, washout slope	Lower maximum slope in TN than in non-TN.
Song et al., 2019 [52]	85 (50 L, 25 HER2, 10 TN)	Peak enhancement, percent volume of enhancement, total enhancing lesion volume	Higher peak enhancement and total enhancing lesion volume in the high-Ki-67 group than those in the low-Ki-67 group.
Yuan et al., 2019 [53]	196 (148 L, 30 HER2, 18 TN)	TIC, early enhancement rate	TIC type positively correlated with positive expression of HER2.
Dilorenzo et al., 2019 [54]	82 (6 LA, 56 LB, 4 HER2)	BPE	Among patients with mild BPE, luminal B tumours were more common. Among patients with marked BPE, TN cancers were more common.
Li et al, 2019 [55]	164	BPE	BPE was positively associated with positive ER status.
Sun et al., 2020 [56]	145 (28 LA, 56 LB, 37 HER2, 24TN)	Ktrans, Kep, Ve, IAUGC60	Higher 5th percentile of the Ktrans, IAUC60, and Ve in the high Ki-67 group.
Matsuda et al., 2020 [57]	50 (50 L)	T1, T2	Higher SD of T1 and T2 after contrast injection in the high Ki-67 group.
Shin et al., 2020 [58]	238 (198 L, 14 HER2, 26 TN)	TTE, maximum slope, SER	Shorter TTE in HER2-positive group than in the HER2-negative group. Shorter TTE in the high Ki-67 group than in the low Ki-67 group.
Onishi et al., 2020 [59]	125 (107 L, 5 HER2, 12 TN)	TTE, maximum slope	Shorter TTE in TN or HER2 subtype compared to L.
Yamaguchi et al., 2021 [60]	97 (69 LA, 14 LB, 5 HER2, 9 TN)	Maximum slope	Maximum slope correlated with Ki-67.
Du et al., 2021 [61]	200 (41 LA, 98 LB, 25 HER2, 36 TN)	Ktrans, Kep, Ve	Higher Ktrans and Kep in HER2 subtype.
Pelissier et al., 2021 [62]	150 (30 LA, 30 LB, 30 HER2, 30 TN, 30 ILC)	Maximum slope	Lower maximum slope in LA.
You et al., 2021 [63]	142 (12 LA, 113 LB, 17 TN)	BPE	Lower BPE in TN.

Abbreviations: AUC, area under the curve; BPE, background parenchymal enhancement; ca, carcinoma; enhancement ratio = (signal intensity after contrast injection − baseline signal intensity)/baseline signal intensity; ER, oestrogen receptor; HER2, human epidermal growth factor receptor 2; HR, hormone receptor; IAUGC60, initial area under the gadolinium curve after the first 60 s; Kep, outflow rate constant; Ktrans, inflow transfer constant; L, luminal type; LA, luminal-A type; LB, luminal-B type; maximum slope = [(maximum signal − baseline signal) × 100%]/[baseline signal × (peak time − contrast arrival time)]; MTT, mean transit time; PR, progesterone receptor; R2 *, 1/T2 *; SD, standard deviation; SER, signal enhancement ratio = (maximum signal − baseline signal)/(signal at last cycle − baseline signal); T2*, T2* relaxation time; TIC, time intensity curve; TN, triple-negative breast cancer; TTE, time-to-enhancement; Ve, leakage space; washout slope = [(signal at last cycle − maximum signal) × 100%]/[maximum signal × (last cycle time − peak time)].

**Table 2:** Summary of diffusion-weighted imaging findings according to molecular prognostic factors and subtypes.

Author, Year	Number of Breast Ca (Subtypes)	Assessment	ROI	Findings
Surov et al., 2017 [67]	476	ADC		Meta-analysis. No significant correlation between Ki-67 and ADC.
Meyer et al., 2021 [66]	2990	ADC		Meta-analysis. No significant difference in ADC values between subtypes.
Kim et al., 2009 [68]	62	ADC	Solid	No significant correlation between ADC and ER, HER2, or Ki-67.
Jeh et al., 2011 [69]	107	ADC	Solid	Lower ADC for ER-positive than for ER-negative status. Higher ADC for HER2-positive than for HER2-negative status.
Choi et al., 2012 [70]	290	ADC	Solid	Lower ADC for ER-positive than for ER-negative status. Lower ADC in the high-Ki-67 group than in the low-Ki-67 group.
Martincich et al., 2012 [71]	192	ADC	Solid	Lower ADC for ER-positive than for ER-negative status. Highest ADC for the HER2-positive subtype.
Youk et al., 2012 [29]	271 (119 L, 94 HER2, 58 TN)	ADC	Whole	Higher ADC for TN than that of others.
Choi et al., 2012 [70]	118 (89 L)	ADC	Solid	Lower ADC for ER-positive status than for ER-negative status. Higher ADC for HER2-positive status than for HER2-negative status.
Richard et al., 2013 [72]	118 (33 LA, 28 LB, 11 HER2, 37 TN)	ADC	Whole	Lower ADC for ER-positive status than for ER-negative status.
Park et al., 2015 [73]	110	ADC	Solid	Higher ADC in HER2-positive IDC than in HER2-negative IDC.
Baba et al., 2014 [74]	70	ADC	Solid	Higher ADC for HER2-positive than HER2-negative status. Lower ADC for ER-positive than ER-negative status.
Miyake et al., 2014 [75]	89	ADC	Unknown	Relative strong correlation for minimal ADCs between the two readers.
De Felice et al., 2014 [76]	75	ADC	Solid	No significant difference in ADC between the high-Ki-67 and low-Ki-67 groups.
Mori et al., 2015 [77]	86 (42 LA, 44 LB)	ADC	Solid	Lower ADC in LB than in LA.
Liu et al., 2015 [78]	176 (67 LA, 45 LB, 29 HER2, 35 TN)	ADC	Whole	Higher ADCs for TN than those for LA or LB. Higher ADCs for HER2-positive subtype than those of LA or LB.
Li et al., 2015 [34]	52	ADC	Solid	Lower ADC in the high-Ki-67 group than in the low-Ki-67 group.
Molinari et al., 2015 [79]	115 (60 LA, 33 LB, 8 HER2, 14 TN)	ADC	Solid	Lower ADC in the high-Ki-67 group than in the low-Ki-67 group. Lower ADC in LB than in LA.
Kim et al., 2015 [80]	173 (43 LA, 84 LB, 9 HER2, 37 TN)	ADC	Whole	Lower ADC in the high-Ki-67 group than in the low-Ki-67 group. Higher ADC for HER2-positive status than for HER2-negative status.
Sun et al., 2015 [81]	52	ADC	Solid	Kurtosis positively correlated with Ki-67. Diffusivity negatively correlated with Ki-67.
Arponen et al., 2015 [82]	104 (11 TN)	ADC	Solid	ADC correlated with PR, but not with HER2, ER, or Ki-67.
Cho et al., 2016 [83]	50 (29 L, 6 HER2, 15 TN)	ADC, IVIM	Whole	Lower ADCmax and Dtmax for ER-positive status. f and Df, showed correlation with hormonal factor expression.
Karan et al., 2016 [84]	70	ADC	Solid	No significant difference in ADC according to ER or HER2 statuses.
Kato et al., 2016 [85]	98 (46 LA, 34 LB, 5 HER2, 13 TN)	ADC	Solid	Higher ADCmin in LA than in LB.
Kong et al., 2018 [86]	46 (27 L, 9 HER2, 10 TN)	ADC	Solid	No significant difference in ADC according to ER or HER2 status. No significant difference in ADC between TN and non-TN.
Lee et al., 2016 [37]	52 (39 L, 4 HER2, 9 TN)	ADC	Solid	Higher ADC for HER2 positive status than for HER2-negative statuses. Lower ADC for ER-positive than for ER-negative statuses.
Guvenc et al., 2016 [87]	48 (38 L, 10 others)	ADC	Solid	Lower ADC for ER-positive than ER-negative status. No significant difference according to HER2 status.
Kitajima et al., 2016 [88]	216 (153 L, 19 HER2, 44 TN)	ADC	Solid	Lower ADC for high Ki-67 than for low Ki-67. No significant difference in ADC according to ER or HER2 statuses.
Kim et al., 2016 [89]	275 (58 LA, 138 LB, 27 HER2, 52 TN)	ADC, IVIM	Solid	No significant difference in ADC for ER, HER2, or Ki-67 statuses. Lower Dt in the high-Ki-67 group than in the low-Ki-67 group.
Shin et al., 2017 [38]	88 (39 LA, 49 LB)	ADC	Solid	Lower ADC in LB than in LA
Shin et al., 2016 [90]	140 (60 LA, 80 LB)	ADC	Solid	Lower ADC in LB than in LA
Durando et al., 2016 [91]	107 (64 L, 20 HER2, 23 TN)	ADC	Solid	No significant difference between subtypes.
Onaygil et al., 2017 [92]	42 (33 L, 9 others)	DTI	Solid	Higher RD and lower FA, RA, and GA for ER-negative status. Ki-67 significantly negatively correlated with FA, RA, and GA.
Lee et al., 2017 [93]	82 (62 L, 9 HER2, 11 TN)	ADC, IVIM	Unknown	Lower ADC for ER-positive than in ER-negative statuses. Dt 50th, 75th, and 90th percentile metrics reduced for ER-positive status. Dt 75th percentile value is a significant differentiator of tumour subtype and Ki-67.
Yamaguchi et al., 2017 [94]	53 (28 LA, 10 LB, 4 HER2, 11 TN)	ADC	Unknown	FA correlated with Ki-67 and ER.
Suo et al., 2017 [95]	49 (27 L, 22 others)	ADC, IVIM	Solid	α and Df correlated with Ki-67. ADC, Dt, f, DDC, and MD correlated with ER.
Catalano et al., 2017 [39]	21 (6 LA, 8 LB, 7 HER2)	ADC	Unknown	Lower ADC in the low-Ki-67 group than in the high-Ki-67 group.
Choi et al., 2017 [96]	221 (149 L, 72 TN)	ADC	Whole	Higher ADC kurtosis in TN than ER-positive status.
Kawashima et al., 2017 [41]	137 (82 LA, 55 LB)	ADC, IVIM	Solid	Lower Dt and ADC in LB than in LA.
Amornsiripanitch et al., 2018 [97]	107 (38 LA, 44 LB, 25 unknown)	ADC, CNR	Whole	DWI CNR associated with Ki-67.
Zhuang et al., 2018 [98]	80	ADC	Whole	Lower ADCmin in the high-Ki-67 group than in the low-Ki-67 group. Higher ADCmax, and ΔADC in the high-Ki-67 group than those in the low-Ki-67 group.
Fan et al., 2018 [99]	126 (26 LA, 67 LB, 22 HER2, 11 TN)	ADC	Whole	Lower ADC in LB than in HER2 subtype.
Aydin et al., 2018 [100]	61 (50 L, 11 others)	ADC	Solid	Weak negative correlation between ADC and Ki-67. No significant difference in ADC according to HER2, ER, or Ki-67 statuses.
Shen et al., 2018 [101]	71 (14 LA, 28 LB, 14 HER2, 15 TN)	ADC	Solid	Lower ADC in the high-Ki-67 group than in the low-Ki-67 group
Incoronato et al., 2018 [45]	49 (13 LA, 29 LB, 4 HER2, 3 TN)	ADC	Unknown	Lower ADC in LB than in non-L.
Ozal et al., 2018 [102]	63 (45L, 18 others)	DTI	Solid	Correlation between ER status and MD, HER2 status and RA, Ki-67 and RA, Ki-67 and VR.
Surov et al., 2018 [103]	870	ADC	Unknown	ADC weakly correlated with Ki-67.
Mao et al., 2018 [104]	77	IVIM	Solid	Ki-67 negatively correlated with Dt.
Zhao et al., 2018 [8]	119 (22 LA, 50 LB, 22 HER2, 25 TN)	ADC, IVIM	Unknown	Higher Df in non-L than in L. TN showed increased Df and f and decreased Dt compared to other subtypes.
Suo et al., 2019 [105]	134 (27 LA, 70 LB, 17 HER2, 20 TN)	ADC	Solid	ADC decreased for ER-positive, PR-positive, and HER2-negative statuses.
Kim et al., 2018 [106]	187 (112 LA, 75 LB)	ADC	Solid	ADC not significantly correlated with Ki-67.
Song et al., 2019 [52]	85 (50 L, 25 HER2, 10 TN)	ADC, IVIM	Solid	No significant associations between IVIM parameters and prognostic factors.
Huang et al., 2019 [107]	46	ADC	Solid	ADC kurtosis positively associated with Ki-67. Mean diffusivity and ADC negatively correlated with Ki-67.
Montemezzi et al., 2018 [50]	453 (66 LA, 292 LB, 39 HER2, 56 TN)	ADC	Solid	Higher ADC in LA than other subtypes. Higher SD of ADC and ADC95th percentile in TN than those in LA.
Xie et al., 2019 [51]	134 (26 LA, 68 LB, 18 HER2, 22 TN)	ADC	Whole	Higher ADC in TN than other subtypes.
Yuan et al., 2019 [53]	196 (148 L, 30 HER2, 18 TN)	ADC	Solid	No significant difference in ADC for HER2, ER, or Ki-67 status.
Horvat et al., 2019 [108]	107 (71 LA, 13 LB, 4 HER2, 19 TN)	ADC	Solid/Whole	Lower ADC in L than in non-L (Whole). No significant difference in ADC for HER2 or ER status (Solid).
You et al., 2019 [109]	148 (14 LA, 75 LB, 40 HER2, 19 TN)	ADC, IVIM	Unknown	HER2-positive cancers showed higher 5th percentile mean diffusivity in the HR-positive group compared to the HR-negative group.
Surov et al., 2019 [110]	661 (177 LA, 279 LB, 66 HER2, 111 TN)	ADC	Solid	Significant overlap of ADC values among different tumour subtypes. Lower ADC in LB compared to LA and HER2 subtypes.
Choi et al., 2019 [111]	101 (50 L, 18 HER2, 20 TN)	ADC	Whole	ΔADC related to Ki-67, molecular subtype.
Horvat et al., 2019 [112]	91 (49 LA, 8 LB, 11 HER2, 23 TN)	ADC	Solid	Higher ADC in the HER2-positive group than in the HER2-negative group. No significant differences according to ER and PR statuses.
Okuma et al., 2020 [113]	88 (82 L, 6 others)	ADC	Solid	Peritumour/tumour ADC ratio significantly associated with Ki-67 but not ER or HER2 status.
Du et al., 2021 [61]	200 (41 LA, 98 LB, 25 HER2, 36 TN)	ADC	Solid	Lower ADC in L than in non-L. Lower ADC in the high-Ki-67 group than in the low-Ki-67 group.
Morawitz et al., 2021 [114]	56 (9 LA, 36 LB, 1 HER2, 6 TN)	ADC	Unknown	Higher ADC in the HER2-positive than in the HER2-negative groups.
Uslu et al., 2021 [115]	51 (27 L, 13 HER2, 11 TN)	IVIM	Unknown	Df and f higher in HER2 subtype than in TN. Df higher in HER2 subtype than in L.
Iima et al., 2021 [116]	86 (60 L, 26 others)	ADC with diffusion time	Solid	Lower ADCshort and ADC in ER-positive group compared to ER-negative group. Larger rate of ADC change with diffusion time in the high-Ki-67 group than in the low-Ki-67 group.
Tuan Linh et al., 2021 [117]	49 (15 LA, 18 LB, 16 HER2)	ADC	Solid	Lower ADC in the high-Ki-67 group than in the low-Ki-67 group. No correlations between ADC and ER, PR, and HER2.
Guo et al., 2021 [118]	105 (79 L, 26 others)	ADC	Whole	10th percentile ADC predicted HER2 and ER statuses. Skewness predicted the Ki-67 status.
You et al., 2021 [63]	142 (12 LA, 39 LB. 74 HER2, 17 TN)	ADC	Unknown	ADC 95th percentile and ADC kurtosis differed significantly among 4 subtypes, especially between TN and other subtypes.

Meta-analysis is shown first; other studies are arranged in chronological order in which they were published on PubMed. Abbreviations: ADC, apparent diffusion coefficient; ADCmax, maximum ADC; ADCmin, minimum ADC; ADCshort, ADC values at short diffusion times; ΔADC, ADCmax – ADCmin; ca, carcinoma; CNR, contrast-to-noise ratio; DDC, distributed diffusion coefficient; Df, fast components of diffusion or pseudodiffusion coefficient; Dt, true diffusion or slow low component of diffusion; Dtmax, maximum Dt; DTI, diffusion tensor imaging; ER, oestrogen receptor; f, fraction of fast diffusion; FA, fractional anisotropy; GA, geodesic anisotropy; HER2, human epidermal growth factor receptor 2; HR, hormone receptor; IDC, invasive ductal carcinoma; IVIM, intravoxel incoherent motion; L, luminal type; LA, luminal-A type; LB, luminal-B type; MD, mean diffusivity; PR, progesterone receptor; RA, relative anisotropy; RD, radial diffusivity; ROI, region of interest; Solid; only solid portion and exclude necrosis, haemorrhage; TN, triple-negative; Unknown, no description of ROI placement method; VR, volume ratio; Whole, whole lesion; α, anomalous exponent term characterizing the deviation from the monoexponential behaviour (0 ≤ α ≤ 1).

**Table 3:** Summary of relaxation time findings according to molecular prognostic factors and subtypes.

Author, Year	Number of Breast Ca (Subtypes)	Assessment	Findings
Liu et al., 2013 [124]	104	R2 *	R2 * weakly correlated with Ki-67 expression
Seo et al., 2017 [122]	92	T2 *	No significant difference in T2 * according to ER or HER2 status.
Matsuda et al., 2020 [57]	50	T1, T2, PD	No significant difference in T1, T2, or PD between the high-Ki-67 group and the-low Ki-67 groups.
Du et al., 2021 [61]	200 (41 LA, 98 LB, 25 HER2, 36 TN)	T1, T2, PD	Higher T1 and T2 in the HR-negative group than in the HR-positive group. Higher T1 and T2 in the high-Ki-67 group than in the low-Ki-67 group.
Li et al., 2021 [123]	122	T1, T2, PD	Higher T2 in the ER-negative group than in ER-positive group. Higher PD in HER2 -positive IDC than in HER2 -negative IDC. The T2 values of the TN, LB, and LA types are arranged in descending order.

Abbreviations: ca, carcinoma; ER, oestrogen receptor; HR, hormone receptor; HER2, human epidermal growth factor receptor 2; IDC, invasive ductal carcinom LA, luminal-A type; LB, luminal-B type; PD, proton density; R2 *, 1/T2 *; T2 *, T2 * relaxation time; TN, triple-negative.

**Table 4:** T Summary of magnetic resonance spectroscopy finding according to molecular prognostic factors and subtypes.

Author, Year	Number of Breast Ca (Subtypes)	Assessment	Findings
Chen et al., 2008 [30]	90	Cho concentration	No significant difference of Cho for ER status.
Sah et al., 2012 [126]	151	Cho concentration	Lower Cho in TN than in non-TN.
Montemezzi et al., 2018 [19]	453 (66 LA, 292 LB, 39 HER2, 56 TN)	Cho SNR	Higher Cho SNR in TN tumours.
Galati et al., 2019 [127]	102 (30 LA, 58 LB, 14 TN)	Cho SNR	Significant association between the presence of Cho peak and higher Ki-67.

Abbreviations: ca, carcinoma Cho, choline; ER, oestrogen receptor; HER2, human epidermal growth factor receptor 2; LA, luminal-A type; LB, luminal-B type; SNR, signal-to-noise ratio; TN, triple-negative.

## Data Availability

The datasets generated during and/or analysed during the current study can be found here: https://mfr.osf.io/render?url=https://osf.io/kzf7q/?direct%26mode=render%26action=download%26mode=render, last accessed on 20 March 2022.
